# An innovative approach to identifying learning needs for intrinsic CanMEDS roles in continuing professional development

**DOI:** 10.1080/10872981.2018.1497374

**Published:** 2018-07-16

**Authors:** Meghan McConnell, Ada Gu, Aysha Arshad, Arastoo Mokhtari, Khalid Azzam

**Affiliations:** a Department of Innovation in Medical Education, University of Ottawa, Ottawa, Canada; b Department of Anesthesiology and Pain Medicine, University of Ottawa, Ottawa, Canada; c Faculty of Health Sciences, McMaster University, Hamilton, Canada

**Keywords:** Intrinsic CanMEDS roles, continuing professional development, learning needs

## Abstract

**Context**: The CanMEDS framework promotes the development of competencies required to be an effective physician. However, it is still not well understood how to apply such frameworks to CPD contexts, particularly with respect to intrinsic competencies.

**Objective**: This study explores whether physician narratives around challenging cases would provide information regarding learning needs that could help guide the development of CPD activities for intrinsic CanMEDS competencies.

**Methods**: We surveyed medical and surgical specialists from Southern Ontario using an online survey. To assess perceived needs, participants were asked, ‘Describe three CPD topic you would like to learn about in the next 12 months’. To identify learning needs that may have arisen from problems encountered in practice, participants were asked, ‘Describe three challenging situations encountered in the past 12 months.’ Responses to the two open-ended questions were analyzed using thematic content analysis.

**Results**: Responses were received from 411 physicians, resulting in 226 intrinsic CanMEDS codes for perceived learning needs and 210 intrinsic codes for challenges encountered in practices. Discrepancies in the frequency of intrinsic roles were observed between the two questions. Specifically, Leader (28%), Scholar (43%), and Professional (16%) roles were frequently described perceived learning needs, as opposed to challenges in practice (Leader: 3%; Scholar: 2%; and Professional: 8%. Conversely, Communicator 39%, Health Advocate 39%, and to a lesser extent Collaborator 11%) roles were frequently described in narratives surrounding challenges in practice, but appeared in <10% of descriptions of perceived learning needs (Communicator: 4%; Health Advocate 6%; Collaborator: 3%).

**Conclusion**: The present study provides insight into potential learning needs associated with intrinsic CanMEDS competencies. Discrepancies in the frequency of intrinsic CanMEDS roles coded for perceived learning needs and challenges encountered in practice may provide insight into the selection and design of CPD activities.

## Introduction

Lifelong learning is an essential part of being a responsible health-care provider. Practicing clinicians must participate in various continuing professional development (CPD) activities in order to maintain and/or enhance competence within their clinical and professional roles [1]. Over the past two decades, there has been an evolution in what it means to be a competent physician [2–5]. Consequently, CPD has expanded its focus beyond traditional medical topics to include various personal and professional competencies necessary for high-quality practice, such as teamwork, communication, managerial skills, professionalism, and teaching skills [6]. This expanded focus is complemented by current shift toward competency-based frameworks, which views clinical competence as more than simple mastery of medical knowledge [7]. For example, the CanMEDS Framework [2] identifies seven ‘roles’ required of a competent medical practitioner: medical expert, collaborator, communicator, health advocate, scholar, professional, and leader. While the latter roles of were initially referred to as ‘nonmedical expert’ competencies, they have since been renamed as ‘intrinsic’ roles to emphasize their importance in meeting the needs of patients, communities and populations served by physicians [8].

Despite the widespread popularity of the CanMEDS Framework, it is still not well understood how to apply such models to CPD activities, particularly with respect to the intrinsic competencies. When selecting CPD activities, ‘physicians tend to focus on their clinical knowledge and skills, rather than other important competencies such as communication, teamwork, practice improvement, and lifelong learning’ ([9], p. 1335). Part of the reason for this focus may be related to a lack of clarity regarding such personal and professional competences. Indeed, researches in academic settings have found that physicians do not fully understand the basic constructs underlying intrinsic competencies [10–13]. For example, faculty members often avoid assessing intrinsic CanMEDS roles, particularly those related to collaborator, leader, health advocate, and scholar, which suggests that individuals may not understand the constructs underlying such roles [11]. Furthermore, physicians do not effectively assess their own professional development needs [14], and such difficulties in self-assessing are likely magnified when it comes to intrinsic competencies, due to poor understanding of these constructs.

For these reasons, CPD programs face challenges in creating educational activities that facilitate the development of intrinsic competencies. While prior studies have examined educational needs related to personal and professional abilities in CPD contexts [9,15–17], these studies have relied on quantitatively based needs assessment surveys to quantify the perceived importance of and interest in such activities. While such approaches are certainly an important step in identifying CPD learning needs, the results from such surveys are limited by the extent to which physicians can accurately assess their learning needs. Given that (a) physicians are not particularly effective at self-assessing gaps in their knowledge and abilities[14], and (b) they do not fully understand the constructs relating to intrinsic competencies [10–13], CPD providers need to find alternative approaches to identify learning needs as they pertain to personal and professional competencies. Since many of physicians learning needs arise from problems arising in practice, we were interested in whether physicians’ descriptions of challenges encountered in practice could provide insight into learning needs pertaining to intrinsic competencies.

The aim of this study was to explore whether physician narratives around challenging cases would provide information regarding intrinsic learning needs that could help guide the development of CPD activities. The research questions were

1. How frequently do medical and surgical specialists identify intrinsic CanMEDS competencies when asked to describe areas for future CPD topics/activities (e.g., perceived learning needs)?
2. How frequently are intrinsic CanMEDS competencies described in narratives describing challenges encountered in practice?

## Methods

Data for present study were collected using an online survey distributed through the Continuing Health Sciences Education Program, McMaster University (Hamilton, ON). During a 6-month time period, recruitment emails were sent to a database of medical and surgical specialists in the Hamilton, Halton, and Niagara regions. Individuals who agreed to participate were sent a second e-mail containing a link to the online survey and all responses were de-linked from e-mail addresses to ensure anonymity of respondents. Participants were entered into a draw for a chance to win one of six prizes of $250. Data for this study were collected as part of a larger online needs assessment survey as part of an ongoing program evaluation of CPD activities, and as a result, the Hamilton Integrated Research Ethics Board considered the study quality improvement and determined that it did not require review by the research ethics board.

Two open-ended questions were used to examine intrinsic roles in practicing clinicians using their own language. To assess perceived needs, participants were asked to ‘Describe three CPD topic you would like to learn about in the next 12 months’. To identify learning needs that may have arisen from problems encountered in practice, participants were asked to ‘Describe three challenging cases or situations encountered in the past 12 months.’ Given that physicians have varying understanding of the constructs underlying intrinsic competencies[10–13], we refrained from asking respondents to describe learning needs and challenges pertaining to these specific competencies. Rather, our aim was to elicit authentic descriptions of learning needs and challenges encountered in practice across a wide range of specialists. Demographic information was also collected through the survey, including gender, years in practice, location of practice (inner city, suburban, small town, rural, geographically isolated, or other), type of practice (individual practice, group practice, community hospital, teaching hospital or other), and specialty.

Qualitative content analysis was used to analyze narrative responses obtained from the open-ended questions. While content analysis refers to a number of different methods used to analyze textual information [18], the present study defined content analysis as ‘a research method for the subjective interpretation of the context of text data through the systematic classification process of coding and identifying themes or patterns’([19], p. 1278). As a first step, one member of the research team (MM) made herself familiar with the data by reading and rereading all written comments for both questions. The dataset included 1050 comments to the question, ‘Describe three CPD topics you would like to learn about in the next 12 months?’ (henceforth referred to as ‘perceived learning needs’) and 908 comments to the question, ‘Describe up to three challenging cases you’ve encountered in the past 12 months’ (henceforth referred to as ‘challenges in practice’). The researcher purposefully excluded all comments that contained responses that could be thematically analyzed (e.g., ‘on maternity leave’, ‘I have nothing to comment’), which included 26 (2.5%) responses for perceived learning needs and 16 (1.8%) responses for challenges in practice.

The final dataset included 1024 written comments describing perceived learning needs and 892 comments documenting challenges in practice. These comments were then reviewed individually by three members of the research team [AG, AM, MM], who coded the responses based on the seven CanMEDS roles of medical expert, collaborator, leader, scholar, communicator, professional, health advocate, and scholar. If a response included more than one CanMEDS role within a single entry, it was separated accordingly into discrete statements. After this initial coding, the entire research team met face-to-face to review the findings and discuss any discrepancies that may have arisen during the coding process, which accounted for less than 5% of the codes. The 1024 comments on perceived learning needs resulted in 1095 codes that mapped onto at least one CanMEDS role, with approximately one-fifth of the comments (*n* = 226; 20.8%) coded to at least one intrinsic CanMEDS role. For the 892 comments describing challenges in practice, a total of 1042 codes that mapped onto at least one CanMEDS role. Once again, approximately one-fifth of the comments (*n* = 210; 20.3%) coded to at least one intrinsic CanMEDS role. After this preliminary analysis, two members of the research team [MM, AG] then conducted a second thematic analysis, focusing only on comments that had at least one intrinsic CanMEDS role. Using a constant comparison strategy, the thematic categories were continually revisited in iterative rounds of discussions[20]. There was high level of agreement between the two raters at this stage of the content analysis (Cohen’s kappa = 0.92). The percentage of comments for each of the six intrinsic CanMEDS roles was determined and compared descriptively for both perceived learning needs and challenges encountered in practice.

## Results

### Participant demographics

Comments were obtained from 411 physicians. As illustrated in Table 1, a little over half of these respondents were male (*n* = 321; 56%) and the majority reported practicing in urban locations (*n* = 315; 77%). Majority of physicians belonged to medical specialties (e.g., internal medicine, pediatrics, psychiatry, etc.; *n* = 296; 72%) relative to surgical specialties (e.g., general surgery, anesthesiology, OBGYN; *n* = 115; 28%). With regards to their years in practice, more than a quarter of the respondents had been practicing medicine for more than 25 years (*n* = 109; 27%), followed by respondents who had been practicing for 1–5 years (18%), 6–10 years (*n* = 74; 14%), 11–15 years (*n* = 59; 13%), 16–20 years (*n* = 55; 10%), and lastly, a few respondents reported being in their first year of practice (*n* = 29; 7%).

**Table 1.** Overall demographic description of study participants.

Responses		*N* (%)
Gender		
	Male	231 (56)
	Female	162 (39)
	Prefer not to answer	18 (4)
Geographic location		
	Geographically isolated	3 (1)
	Small town	42 (10)
	Suburban	41 (10)
	Urban	315 (77)
	Other	10 (2)
Specialty		
	Medical	296 (72)
	Surgical	115 (28)
Years in practice		
	First year in practice	28 (7)
	1–5 years	74 (18)
	6–10 years	59 (14)
	11–15 years	55 (13)
	16–20 years	44 (11)
	21–25 years	42 (10)
	>25 years	109 (27)
**Total**		**411 respondents**

### Responses to open-ended questions

**Figure 1.**
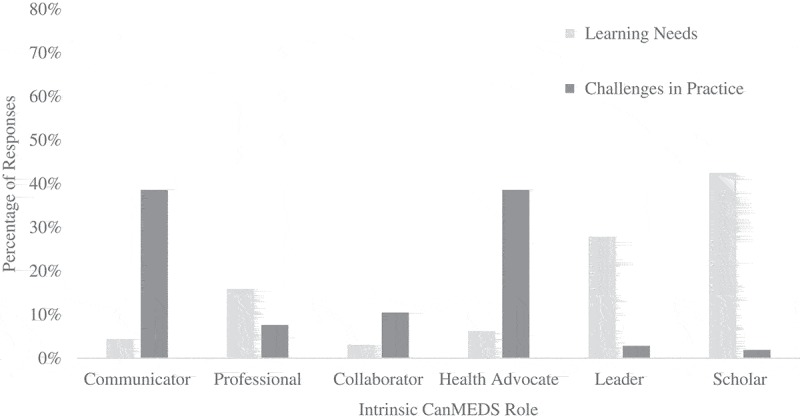
Content analysis of intrinsic CanMEDs roles obtained from response to open-ended questions aimed at identifying perceived learning needs and challenges encountered in clinical practice.

The response to the open-ended questions (e.g., perceived learning needs and challenges in practice) were organized by intrinsic CanMEDS role. Figure 1 illustrates the percentage of intrinsic CanMEDS roles that were coded within each question format. Recurrent themes were identified within each intrinsic role, and we include representative quotes to illustrate our findings (see Table 2).

**Table 2.** Summary of major themes for each intrinsic CanMEDS role that emerged from comments with illustrative quotes.

	Learning needs	Challenges in practice
**Communicator**	***Theme: Difficult conversations*** ‘Difficult code-status discussions’ ‘Dilemma in clinical communication’ ‘Dealing with difficult patients’	***Theme: Personality attributes of patients and/or families*** ‘Difficult patients, i.e., verbally abusive, threatening harm to others’ ‘Management of hostile patients’ ‘Complex family making it difficult to treat’ ***Theme: Unrealistic expectations or requests*** ‘Demands of patients/families for tests/treatments not available’ ‘Dealing with families unrealistic expectations for terminal illness’ ***Theme: Patient noncompliance*** ‘Non-compliant patient failing to attend appointments’ ‘DM pt [*sic*] refuses to take medication’ ***Theme: Balancing multiple perspectives*** ‘Balancing family wishes and patient wishes with a patient with Huntington’s’ ‘Disagreement in management between parents’
**Professional**	***Theme: Physician well-being*** ‘Maintaining motivation and preventing burnout’ ‘Physician self-care’ ‘Managing stress, time and work-life balance’ ***Theme: Ethical and medicolegal aspects of practice*** ‘Ethics of physician assisted suicide’ ‘Ethics and end of life care’ ‘Occupational medicine and the law’	***Theme: Ethical and medicolegal aspects of practice*** ‘Ethics of physician assisted suicide’ ‘Ethics of surrogacy’ ‘Medico-legal frameworks governing practices’ ‘Highly litigious patient making unreasonable requests’
**Collaborator**	***Theme: Interprofessional health care*** ‘Interprofessional collaboration’ ‘Integrated health care’	***Theme: Conflict resolution and management*** ‘Disagreeing with management of consultant’ ‘Colleague power struggles’ ‘Interprofessional conflicts’ ***Theme: Transitions of care*** ‘When and where to transfer an epilepsy patient to adult care’ ‘Elderly patient with cardiac disease and fractured hip – come to operating room with medical clearance but not full cardiac workup’ ‘Patients sent for admission who do not need admission’
**Health Advocate**	***Theme: Promoting healthy behaviors*** ‘Healthy lifestyle and metabolic factors’ ‘Integration of socioeconomic improvements and health’ “ACL injury prevention ***Theme: International aspects of health care*** ‘Medicine in the developing world’ ‘Newborn care in developing areas of the world’ ‘International health and surgery’	***Theme: Accessing resources*** ‘Patients cannot buy meds’ ‘pt [*sic*] with ALS and chooses invasive ventilation and struggle to get resources to support him’ ‘Lack of resources for palliative patients in the community’ ***Theme: Social determinants of health*** ‘Multisystem ill patient with complex social situation’ ‘Complicated psychosocial environment’ ***Theme: Promoting healthy behaviors*** ‘Promoting weight loss’ ‘Getting patients to ambulate’
**Leader**	***Theme: Leadership skills*** ‘Leadership skills development’ ‘Medical leadership and administration’ ***Theme: Practice management*** ‘Business administration’ ‘Running an office’ ***Theme: Budgeting and financing*** ‘Health care insurance’ ‘Billing practices for surgeons’	***No clear themes, example quotes:*** ‘Bringing about change’ ‘Resources in hospitals’ ‘Raising awareness of overuse in antimicrobials’
**Scholar**	***Theme: Educational practices*** ‘Giving effective feedback/evaluation to learners’ ‘Teaching strategies within a busy clinical practice’ ‘Curriculum development for medical students’ ***Theme: Research and scholarship*** ‘Network meta-analysis’ ‘Advanced RCTs’ ‘Successful grant applications’ ***Theme: Evidence informed decision making*** ‘Implementation of clinical practice guidelines’ “Establishing guidelines of axillary lymph node management for breast cancer patients	***Theme: Research and scholarship*** ‘Conducting multinational RCTs’ ‘Grants preparation’

#### Communicator

Less than 5% (*n* = 10; 4.4%) of the perceived learning needs responses were coded to the CanMEDS Communicator role. These responses ranged in content, although three responses described a need for CPD topics related to difficult communication topics (see Table 2).

In contrast, over one-third (*n* = 81; 38.6%) of the responses describing challenges in practice aligned with the Communicator role. Within these responses, four major themes emerged. The first related to *Personality Attributes of Patients and/or Family Members*, which included responses describing challenges arising from perceived negative temperament or demeanor of patients and/or their family members. The second theme included *Unrealistic Expectations or Requests*, which described challenges that arose due to expectations from patients and/or family members that were perceived as unrealistic or inappropriate. The third theme described *Noncompliance*, which included challenges associated with the ‘management of noncompliant patients’. Lastly, the fourth theme included *Balancing multiple perspectives*, which described situations where there were multiple, and sometimes conflicting, opinions between patients and their families, or between individual family members with regards to the care of their family member (e.g., child, parent).

#### Professional

A total of 36 (15.9%) perceived learning need responses aligned with the Professional role. From these comments, two professionalism-related themes emerged. The first described *Physician Well-Being*, which included responses regarding both physician wellness and achieving some level of balance between professional and personal responsibilities. The second theme described *Ethical and Medicolegal Aspects of Medicine*, which included generic statements (e.g., ‘ethics’) as well as specific ethical practices such as physician-assisted suicide.

Sixteen (7.6%) responses describing challenges encountered in practice were coded to the Professional role. Just over half of these responses described challenges related to *Ethical and Medicolegal Aspects of Medicine*, which included comments on ethical practices within medicine, as well as specific ethical/legal encounters with patients (e.g., ‘complicated court assessment of psychotic person facing serious charges’).

#### Collaborator

The Collaborator role represented the fewest number of responses to perceived learning needs (*n* = 7; 3.1%). These seven comments ranged in content, although two responses described components of *Interprofessional Healthcare* (see Table 2).

A total of 22 (10.5%) responses describing challenges encountered in practice aligned with the Collaborator role. Nearly half of these responses described challenges arising from *Conflict Resolution and Management*, such as differences in opinions among health-care professionals, as well as intra- and inter-professional conflicts. A few responses also referred to *Transitions of Care*, which described challenges of ‘when and where’ to transfer of patient care, and also included scenarios where the challenge originated from what was perceived as inappropriate transfer of care.

#### Health advocate

A total of 14 (6.2%) of responses to perceived learning needs aligned to the Health Advocate role. About a third of these responses described *Promoting Healthy Behaviors*, which included comments describing various ways to promote patient health well-being. Several comments also described a need to learn about *International Aspects of Health Care*, particularly within developing areas of the world.

In contrast, the Health Advocate role was frequently coded in responses describing challenges encountered in practice (*n* = 81; 38.6%). Three major themes emerged from the data. First, *Accessing Resources* included challenges in accessing both individual and community resources. Second, *Social Determinants of Health* included comments describing challenges associated with social factors affecting patient care. Lastly, *Promoting Healthy Behaviors* included comments related to encouraging healthy behaviors in patients.

#### Leader

A total of 63 (27.9%) of perceived learning need responses aligned with the Leader role. From these responses, three themes emerged [1]: *Leadership Skills*, which included responses related to the development of leadership skills; [2] *Practice management*, which described learning needs around practice management and administrative duties [3]; and *Budgeting and Financing*, which included responses related to financial aspects of clinical practice, such as billing and health insurance developments.

In contrast, only six (2.9%) responses describing challenges in practice aligned with the Leader Role. These responses ranged in content and did not appear to cluster into any meaningful themes.

#### Scholar

A total of 96 (42.5%) of perceived learning need responses were coded to the Scholar Role. Three major subthemes were identified. The first theme described learning needs relating to *Educational Practices*, included responses describing the development of various educational skills, including assessing learners and providing feedback, developing teaching skills, and creating education curricula. The second theme described *Research and Scholarship*, included responses about specific research methodologies, as well as responses related to obtaining research funding. The final theme related to *Evidence-Informed Decision Making*, which included responses specific to the implementation/development of clinical practice guidelines.

The Scholar role represented the fewest number of responses to challenges encountered in practice (*n* = 4; 1.9%). These responses ranged in content, although two described challenges related to *Research and Scholarship*.

## Discussion

The present study provides insight into potential educational needs mapped onto Intrinsic CanMEDS competencies within an Ontario sample of medical and surgical physicians. To our knowledge, this study is the first to systematically quantify and content analyze written responses describing areas for future CPD topics/activities (e.g., perceived learning needs) and challenges encountered in practice in an attempt to identify educational needs related to intrinsic CanMEDS competencies. While prior research has identified professional and personal learning needs using quantitative surveys [9,15–17], the present study used qualitative content analysis of physicians’ narratives to open-ended questions. These comments provided a richness and authenticity to how these intrinsic roles are constructed in practice that could not have been possible from quantitative data alone.

Responses to both sets of questions elicited responses could be mapped across all intrinsic CanMEDS roles. Several topics areas identified for future CPD topics/activities (e.g., perceived learning needs) intersect previous research findings [9,15,16]. For example, a recent study examining Irish general practitioners found that respondents highly endorsed educational activities related to practice management, ethics, and stress management [15]. Relatedly, Phitayakorn et al. reported that a high percentage of surgical educators requested further training in research techniques, such as finding grant funding, designing research studies, and performing data analyses [16]. These results align with several themes identified in the present study, including professional constructs such as physician well-being and ethical aspects of practice; Leader constructs such as practice management; and Scholar constructs such as research and scholarship practices.

While both sets of questions contained responses that aligned with an Intrinsic CanMEDS role, differences in the frequency of these intrinsic competencies were observed between the two questions. For example, Leader and Scholar, and to a lesser extent Professional, were coded more frequently in physicians’ descriptions of desired future CPD activities relative to descriptions of challenges encountered in their practice. Conversely, Communicator and Health Advocate, and to a lesser extent Collaborator, were reported more frequently in physicians’ descriptions of challenges encountered in their practice as opposed to descriptions of desired future CPD activities. Such discrepancies suggest that narratives describing challenges encountered in practice may provide information not obtained by asking individuals to explicitly identify their learning needs. In this way, qualitatively analyzing challenges may provide insight into unperceived learning needs in personal and professional competencies.

Such discrepancies could have implications in the selection and design of CPD activities. At the individual level, analysis of challenges encountered in practice may help physicians identify and recognize potential educational gaps and make informed decisions when choosing CPD activities. For CPD programs, descriptions of challenges encountered in practice may provide an innovative way to develop educational content that meets the learning needs of physicians. For example, in the present study, dealing with difficult patients and families was frequently mentioned in narratives of challenging encounters. Based on such findings, CPD providers could develop activities that provide physicians with knowledge, skills, and resources in how to deal with potentially challenging temperaments and demeanors of patients and/or their family members. This is not to say that entire activities should necessarily be devoted solely to this topic – more research would certainly be needed to warrant this approach. Instead, current CPD activities could be modified to include information and activities on dealing with ‘difficult’ patients within a given clinical context.

This study does have its limitations. The data were predominantly from one region, which limits the generalizability of our findings. Additionally, because the study took place at an academic medical center, some responses may not generalize to physicians in community-based practices. For example, academic physicians have higher teaching loads and research responsibilities as opposed to community-based physicians, and so learning needs associated with enhancing educational practices and research skills may not generalize to physicians who are not affiliated with an academic center. Another limitation of this study is the presumption that the intrinsic roles identified in descriptions of challenging encounters can be addressed through CPD activities. This simply may not be the case. For example, it is possible that dealing with ‘difficult’ patients and/or families is challenging not because physicians’ lack the necessary communication skills, but rather, simply find such exchanges to be emotionally and/or cognitively demanding. While the use of open-ended survey questions allowed us to obtain a large sample of textual responses, it did not allow us to investigate the reasons underlying these responses. For this reason, more research is needed to further explore the factors underlying these challenging encounters to determine how (and if) CPD activities can curtail such challenges.

To conclude, this study has described a novel approach to identifying learning needs relating to intrinsic CanMEDS roles. While the assessment of medical expertise is an essential component of medical education, incorporating personal and professional competencies such as teamwork, communication, professionalism, and managerial roles is also vital to effective CPD activities. Through qualitative content analysis of comments provided by physicians from a range of specialties and years in practice, the present study provides insight into potential educational needs associated with intrinsic CanMEDS competencies. These findings should be further investigated in a larger, more geographically varied sample of physicians to determine whether narratives of challenges encountered in practice can help identify gaps in personal and professional competencies and can be used by CPD providers to develop more targeted and timely programs.

## References

[1] Campbell C, Silver I, Sherbino J, et al Competency-based continuing professional development. Med Teach. 2010;32:657–8. 20662577 10.3109/0142159X.2010.500708

[2] Frank J. The CanMEDS 2005 physician competency framework: better standards, better physicians, better care. Ottawa, ON: The Royal College of Physicians and Surgeons of Canada; 2005.

[3] Rubin P, Ranchi-Christopher D New edition of tomorrow’s doctors. Med Teach. 2002;24:368–369. 12193317 10.1080/0142159021000000816

[4] Batalden P, Leach D, Swing S, et al General competencies and accreditation in graduate medical education. Health Aff. 2002;21:103–111. 10.1377/hlthaff.21.5.103 12224871

[5] Simpson J, Furnace J, Crosby J, et al The Scottish doctor - learning outcomes for the medical undergraduate in Scotland: a foundation for competent and reflective practioners. Med Teach. 2002;24:136–143. 12098432 10.1080/01421590220120713

[6] Chan K Medical education: from continuing medical education to continuing professional development. Asia Pac Fam Med. 2002;1:88–90.

[7] Harden R Outcome-based education: the ostrich, the peacock and the beaver. Med Teach. 2007;29:666–671. 18236254 10.1080/01421590701729948

[8] Sherbino J, Frank J, Flynn L, et al “Intrinsic roles” rather than “armour”: renaming the “non-medical expert roles” of the CanMEDS framework to match their intent. Adv Heal Sci Educ. 2011;16:695–697. 10.1007/s10459-011-9318-z 21850502

[9] Cook D, Blachman M, Price D, et al Professional development perceptions and practices among US physicians: a cross-specialty national survey. Acad Med. 2017;92:1335–1345. 28225460 10.1097/ACM.0000000000001624

[10] Puddester D, MacDonald C, Clements D, et al Designing faculty development to support the evaluation of resident competency in the intrinsic CanMEDS roles: practical outcomes of an assessment of program director needs. BMC Med Educ. 2015;15:100. 26043731 10.1186/s12909-015-0375-5 PMC4472249

[11] Sherbino J, Kulasegaram K, Worster A, et al The reliability of encounter cards to assess the CanMEDS roles. Adv Heal Sci Educ. 2013;18:987–996. 10.1007/s10459-012-9440-6 23307097

[12] Renting N, Dornan T, Gans R, et al What supervisors say in their feedback: construction of CanMEDS roles in workplace settings. Adv Heal Sci Educ. 2016;21:375–387. 10.1007/s10459-015-9634-9 PMC4801985 26342599

[13] Renting N, Raat A, Dornan T, et al Integrated and implicit: how residents learn CanMEDS roles by participating in practice. Med Educ. 2017;51:942–952. 28485074 10.1111/medu.13335

[14] Eva K, Bordage G, Campbell C, et al Towards a program of assessment for health professionals: from training into practice. Adv Heal Sci Educ. 2016;21:897–913. 10.1007/s10459-015-9653-6 26590984

[15] Maher B, O’Neill R, Faruqui A, et al Survey of Irish general practitioners’ preferences for continuing professional development. Educ Prim Care. 2018;29:13–21. 28612643 10.1080/14739879.2017.1338536

[16] Phitayakorn R, Salles A, Falcone J, et al A needs assessment of education research topics among surgical educators in the USA. Am J Surg. 2017;213:346–352. 27955883 10.1016/j.amjsurg.2016.11.044

[17] Ringstead C, Hansen T, Davis D, et al Are some of the challenging aspects of the CanMEDS roles valid outside Canada? Med Educ. 2006;40:807–815. 16869928 10.1111/j.1365-2929.2006.02525.x

[18] Vaismoradi M, Turunen H, Bondas T Content analysis and thematic analysis: implications for conducting a qualitative descriptive study. Nurs Heal Sci. 2013;15:398–405. 10.1111/nhs.12048 23480423

[19] Hsieh H, Shannon S Three approaches to qualitative content analysis. Qual Health Res. 2005;15:1277–1288. 16204405 10.1177/1049732305276687

[20] Downe-Wamboldt B Content analysis: method, applications, and issues. Heal Care Women Int. 1992;13:313–321. 10.1080/07399339209516006 1399871

